# Phytochemical composition, antiparasitic and α–glucosidase inhibition activities from *Pelliciera rhizophorae*

**DOI:** 10.1186/s13065-015-0130-3

**Published:** 2015-09-28

**Authors:** Dioxelis López, Lilia Cherigo, Carmenza Spadafora, Marco A. Loza-Mejía, Sergio Martínez-Luis

**Affiliations:** Center for Drug Discovery and Biodiversity, Institute for Scientific Research and Technology Services (INDICASAT), Clayton, P.O. Box 0843-01103, Panama City, Republic of Panama; Department of Biotechnology, Acharya Nagarjuna University, Nagarjuna Nagar, Guntur, 522510 India; Department of Organic Chemistry, Chemistry School, Faculty of Natural Sciences, Exact and Technology, University of Panama, P.O. Box 3366, Panama City, Republic of Panama; Center for Cellular and Molecular Biology of Diseases, Institute for Scientific Research and Technology Services (INDICASAT), Clayton, P.O. Box 0843-01103, Panama City, Republic of Panama; Facultad de Ciencias Químicas, Universidad La Salle, Benjamín Franklin 47, Cuauhtémoc, 06140 Mexico City, Mexico

**Keywords:** α-Glucosidase, *Pelliciera rhizophorae*, Triterpenes, Mangroves, Diabetes

## Abstract

**Background:**

Panama has an extensive mangrove area and it is one of the countries with the highest biodiversity in America. Mangroves are widely used in traditional medicine, nevertheless, there are very few studies that validates their medicinal properties in America. Given the urgent need for therapeutic options to treat several diseases of public health importance, mangrove ecosystem could be an interesting source of new bioactive molecules. This study was designed to evaluate the potential of *Pelliciera rhizophorae* as a source of bioactive compounds.

**Results:**

The present investigation was undertaken to explore the possible antiparasitic potential and α-glucosidase inhibition by compounds derived from the Panamanian mangrove *Pelliciera rhizophorae*. Bioassay-guided fractionation of the crude extract led to the isolation of ten chemical compounds: α-amyrine (**1**), β-amyrine (**2**), ursolic acid (**3**), oleanolic acid (**4**), betulinic acid (**5**), brugierol (**6**) iso-brugierol (**7**), kaempferol (**8**), quercetin (**9**), and quercetrin (**10**). The structures of these compounds were established by spectroscopic analyses including APCI-HR-MS and NMR. Compounds **4** (IC_50_ = 5.3 µM), **8** (IC_50_ = 22.9 µM) and **10** (IC_50_ = 3.4 µM) showed selective antiparasitic activity against *Leishmania donovani*, while compounds **1** (IC_50_ = 19.0 µM) and **5** (IC_50_ = 18.0 µM) exhibited selectivity against *Tripanosoma cruzi* and *Plasmodium falciparum*, respectively. Moreover, compounds **1**–**5** inhibited α-glucosidase enzyme in a concentration-dependent manner with IC_50_ values of 1.45, 0.02, 1.08, 0.98 and 2.37 µM, respectively. Their inhibitory activity was higher than that of antidiabetic drug acarbose (IC_50_ 217.7 µM), used as a positive control. Kinetic analysis established that the five compounds acted as competitive inhibitors. Docking analysis predicted that all triterpenes bind at the same site that acarbose in the human intestinal α-glucosidase (PDB: 3TOP).

**Conclusions:**

Three groups of compounds were isolated in this study (triterpenes, flavonols and dithiolanes). Triterpenes and flavones showed activity in at least one bioassay (antiparasitic or α-glucosidase). In addition, only the pentacyclic triterpenes exhibited a competitive type of inhibition against α-glucosidase.

## Background

Present world is a place of high mortality rates mainly due to severe poverty. High levels of poverty results in malnutrition, overcrowding, bad sanitation and polluted water. These conditions lead to a fertile environment for parasitic diseases and diabetes. A parasitic infection is one of the leading causes of chronic human diseases in most tropical countries. The parasites, including protozoa and helminthes, infect billions of people and can result in blindness, disfigurement, or even death. Efforts to develop vaccines against these pathogens have been prevented by the difficulty of cultivation of parasites in the laboratory, the complexity of their multicellular organization and many species have been developing impressive antigenic variability. At the same time, most treatments involve highly toxic drugs and parasites has greatly increased the drug-resistant and finally, the chemotherapeutic agents used in infected patients have lacked effectiveness [1–3].

Another major cause of mortality is Diabetes mellitus (DM), it was responsible for about 1.5 million deaths in 2012. According to World Health Organization (WHO) forecasts, DM will be the 7th leading cause of death in 2030, and its prevalence has shown to be higher in low and middle-income countries. This disease is known for allowing high sugar levels in human blood, either because insulin production is inadequate or because the body’s cells do not respond properly to insulin, or both. Patients with high blood sugar will typically present many fatal disorders in different organs, including hyperosmolar hyperglycemic nonketotic syndrome, feet and skin complications, amputations, hypertension, retinopathy, neuropathy and diabetic nephropathy [4, 5]. Thus, there is an urgent need to search for novel drugs from several sources, including natural products, to fight global health problems posed by parasitic infections and DM.

In the western hemisphere, mangroves are a natural source that has been poorly explored for biomedical potential. Mangroves are a group of halophytes plants that are developed in the tropical or subtropical areas, functioning as a bridge between the marine and terrestrial habitats. Mangroves are highly adapted to their environment and they are able to deal with many physical stress factors such as strong variation in moisture and salt concentrations, changing tides, and biological stressing factors produced by abundant herbivorous insects [6]. Currently, mangrove plants and their extracts are mainly used by dwellers for medicinal use, especially for the treatment skin infections, tuberculosis, skin wounds, diarrhea, and other uses such as insecticides and piscicides [6–8]. Mangroves are a good source of secondary metabolites such as alkaloids, steroids, flavonoids, saponins, tannins, and triterpenes. Chemical studies using mangrove plants have led to the isolation of over 200 bioactive compounds [8, 9].

Panama has an extensive mangrove area and it is one of the countries with the highest biodiversity in America [6]. Mangroves are widely used in traditional medicine, nevertheless, there are very few studies that validates their medicinal properties in America. Given the urgent need for therapeutic options to treat several diseases of public health importance, mangrove ecosystem could be an interesting source of new bioactive molecules. This study was designed to evaluate the potential of *Pelliciera rhizophorae* as a source of bioactive compounds. Here we report the isolation, identification and bioactivity against three parasites (*Leishmania donovani*, *Plasmodium falciparum* and *Trypanosoma cruzi*) and the modulation of α-glucosidase function of compounds produced by this plant, which is endemic mangrove from Central America.

## Results

### Chemical study

**Fig. 1:**
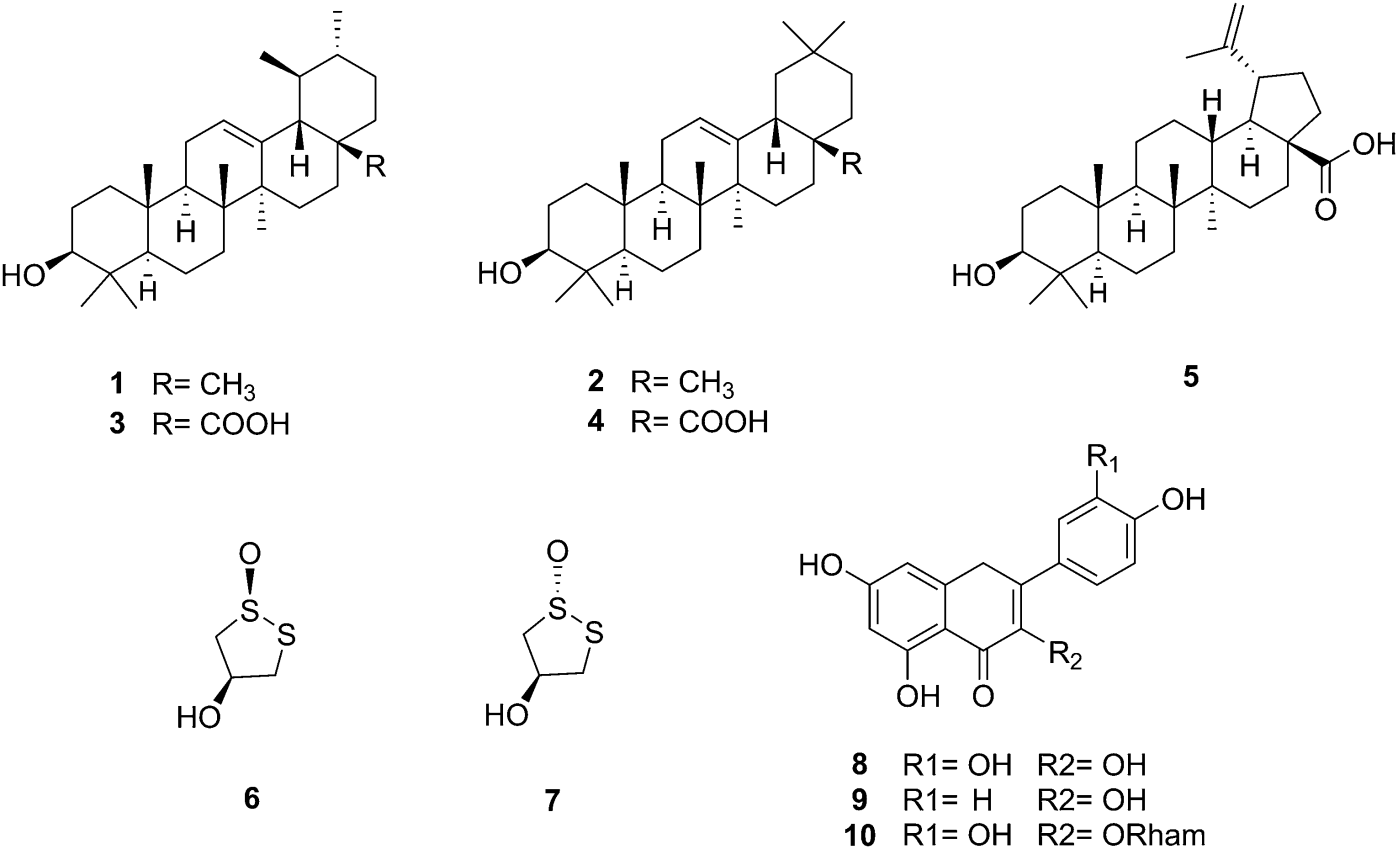
Structures of compounds produced by *Pelliciera rhizophorae*

*Pelliciera rhizophorae* (Pellicieraceae) is an endemic mangrove plant from the Central American coasts. Mangrove leaves were collected in the protected mangrove area of Chame Bay, Panama. In the initial screening (against *P. falciparum*, *T. cruzi* and *L. donovani*), the crude extract did not present antiparasitic activity but it showed good inhibition against α-glucosidase enzyme (82 % of inhibition). Following the protocols of our laboratory we performed a primary fractionation by open column chromatography to afford 37 fractions. All fractions were submitted for bioactivity testing, resulting in fractions IX (70 % growth inhibition (GI) against *T. cruzi*), FXIII (83 % GI against *L. donovani* and 69 % GI against *P. falciparum*), FXXVIII (80 % GI against *L. donovani*, 73 % GI against *P. falciparum* and 67 % GI against *T. cruzi*), and FXXXIII (75 % GI against *L. donovani*) having antiparasitic properties and fractions IX (86 %), FXIII (93 %), and FXXXIII (84 %) showed inhibition of α-glucosidase activity at a concentrations of 10 µg/mL for parasites and 6.25 µg/mL for α-glucosidase. Bioassay-guided fractionation of the active fractions yielded compounds α-amyrine (**1**), β-amyrine (**2**), ursolic acid (**3**), oleanolic acid (**4**), betulinic acid (**5**), brugierol (**6**), iso-brugierol (**7**), kaempferol (**8**), quercetin (**9**) and quercetrin (**10**), which were established by spectroscopic analyses including APCI-HR-MS and NMR (^1^H, ^13^C, DEPT 135, DEPT 90, COSY, NOESY, HMBC and HMQC). The structures (Fig. 1) were corroborated by comparing the spectroscopic and spectrometric data with those previously reported [10–18]. Even though the isolated compounds are quite common compounds, and are found in many other plant species, it should be emphasized that this is the first report about the occurrence of triterpenes, flavonoids and dithiolanes in the genus Pelliciera. Therefore, this plant represents a new source of this type of bioactive substances, which in several studies they have shown beneficial properties for human health.

### Antiparasitic activity

Compounds **3**, **4**, **8**, **9** and **10** displayed biological activity against the amastigotes of *L. donovani* (Table 1). This result was expected mainly because in previous reports ursolic acid showed activity against the promastigotes of *L. donovani* [19], oleanolic acid inhibited the promastigotes of *L. braziliensis* and *L. chagasi* [20], while quercetin, kaempferol and quercetrin displayed a leishmanicidal effect on the amastigote stage of *L. donovani* [19, 21, 22]. Moreover, compounds **3**, **5** and **9** were active against a chloroquine-resistant strain (Indochina W2) of *P. falciparum*. These compounds had previously shown activity against *P. falciparum* chloroquine-sensitive 3D7 (**3**, **5** and **9**) and chloroquine-resistant K1 (**9**) [19, 23]. Finally, compounds **1** and **9** also showed bioactivity against *T. cruzi*, strain Tulahuen, clone C4 (Table 1). These compounds had previously exhibited activity against trypomastigotes of *T. brucei rhodesiense* which causes African trypanosomiasis [24, 25].

**Table 1:** Antiparasitic activity (IC_50_, μM) of the isolated compounds from *Pelliciera rhizophorae*

Compounds	*L. donovani*	*P. falciparum*	*T. cruzi*	*Vero cells*
**1**	I	I	19.0 ± 0.6	I
**2**	I	I	I	I
**3**	2.4 ± 0.1	21.9 ± 0.6	I	64.8 ± 0.3
**4**	5.3 ± 0.2	I	I	140.8 ± 0.5
**5**	I	18.0 ± 0.4	I	131.1 ± 0.3
**6**	I	I	I	I
**7**	I	I	I	I
**8**	22.9 ± 0.2	I	I	154.9 ± 0.6
**9**	12.6 ± 0.2	9.7 ± 0.3	13.0 ± 0.4	I
**10**	3.4 ± 0.1	I	I	I
Anfotericine B	1.0 ± 0.12	ND	ND	ND
Chloroquine	ND	0.09 ± 0.01	ND	ND
Nifurtimox	ND	ND	1.6 ± 0.11	ND

*I* inactive at 10 µg/mL, *ND* not determined

Although the antiparasitic activity of the isolated compounds is not comparable to that of controls, it is important to emphasize two important aspects, first the isolated compounds were evaluated against three different parasites of human importance so it is possible to describe some selectivity in the activity of *P. rhizophorae* components, and second these compounds showed low levels of cytotoxicity. According to this, compounds **1**, **4**, **5**, **8** and **10** have selective activity against one parasite while compound **9** possess broad activity inhibiting the three evaluated parasites. Nevertheless, the main trouble with these compounds will be their low polarity, which gives them aqueous solubility problems, and therefore low absorption and limited bioavailability. Developing analogues with higher polarity and better biological activity would be a viable option to overcome this obstacle.

Infectious diseases, including causative agents of trypanosomiasis, leishmaniasis and malaria, are responsible for a high rate of mortality and morbidity each year in the countries with high levels of poverty. Due to the lack of treatment options, there is an urgent need to discover novel therapeutics options against these neglected tropical diseases. Thus, the discovery of new sources of antiparasitic agents is of great significance, because natural sources are one of the most affordable, especially for people in poor countries.

### α-Glucosidase inhibition evaluation and kinetic studies

Triterpenes **1**–**5** inhibited α-glucosidase enzyme in a concentration-dependent manner with IC_50_ values of 1.45, 0.02, 1.08, 0.98 and 2.37 µM, respectively. All triterpenes were more potent against α-glucosidase than acarbose (positive control, IC_50_ 217.7 µM). Given the structural similarities and biological activity among compounds **1**–**5**, the minor substitutions of the central core (pentacyclic triterpene) does not appear to produce a significant difference in the α-glucosidase inhibition. However, if we compare compound **2** with the other compounds, it can be inferred that presence of a gem dimethyl at position 20 and a methyl in position 28, it confer a potent inhibitory activity to the pentacyclic core.

**Fig. 2:**
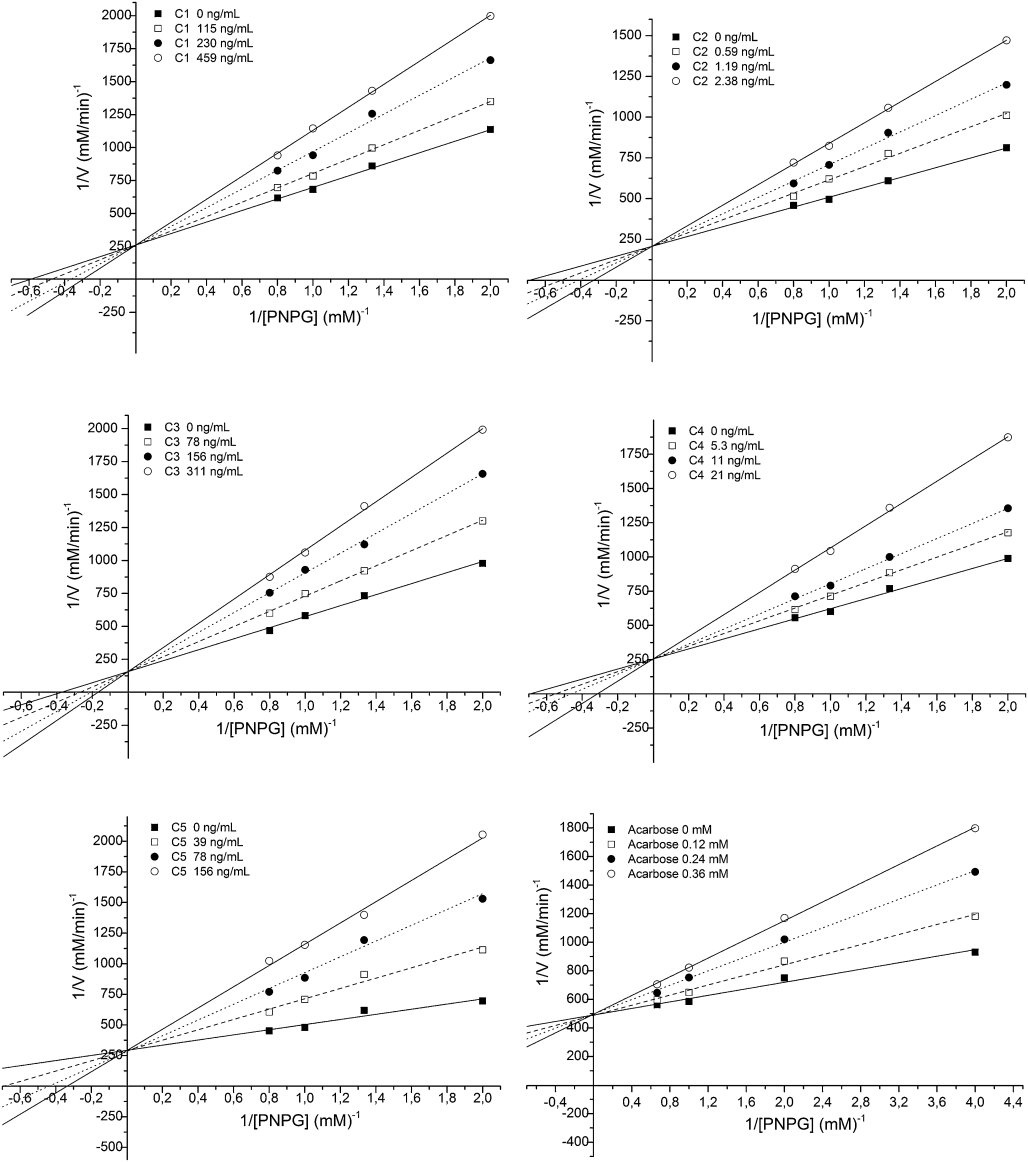
Lineweaver–Burk plots of α-glucosidase inhibition at different concentrations of substrate, compounds **1**–**5** and acarbose

In order to obtain further evidence of the nature of the interaction of compounds **1**–**5** with α-glucosidase kinetic analyses were carried out. Lineweaver–Burk plots [26] were constructed using different concentrations of substrate and triterpenes **1**–**5**. The results in Fig. 2 indicated that **1**–**5** showed typical reversible competitive plots, with series of lines having the same y-intercept as the enzyme without inhibitors. These results suggested that compounds **1**–**5** bind to α-glucosidase or to the substrate-enzyme complex. Acarbose also behaved as competitive inhibitor [27]. These results show that pentacyclic triterpene core is a potent competitive inhibitor of the α-glucosidase enzyme.

With respect to the modulations of α-glucosidase function by compounds **6**–**10**, metabolites **8** (262.2 µM) and **9** (29.8 µM) showed moderate inhibition while **6**, **7** and **10** were inactive, so it can be hypothesized the main α-glucosidase inhibitors produced by *P. rhizophorae* belongs to pentacyclic triterpenoid family.

### Docking study

**Fig. 3:**
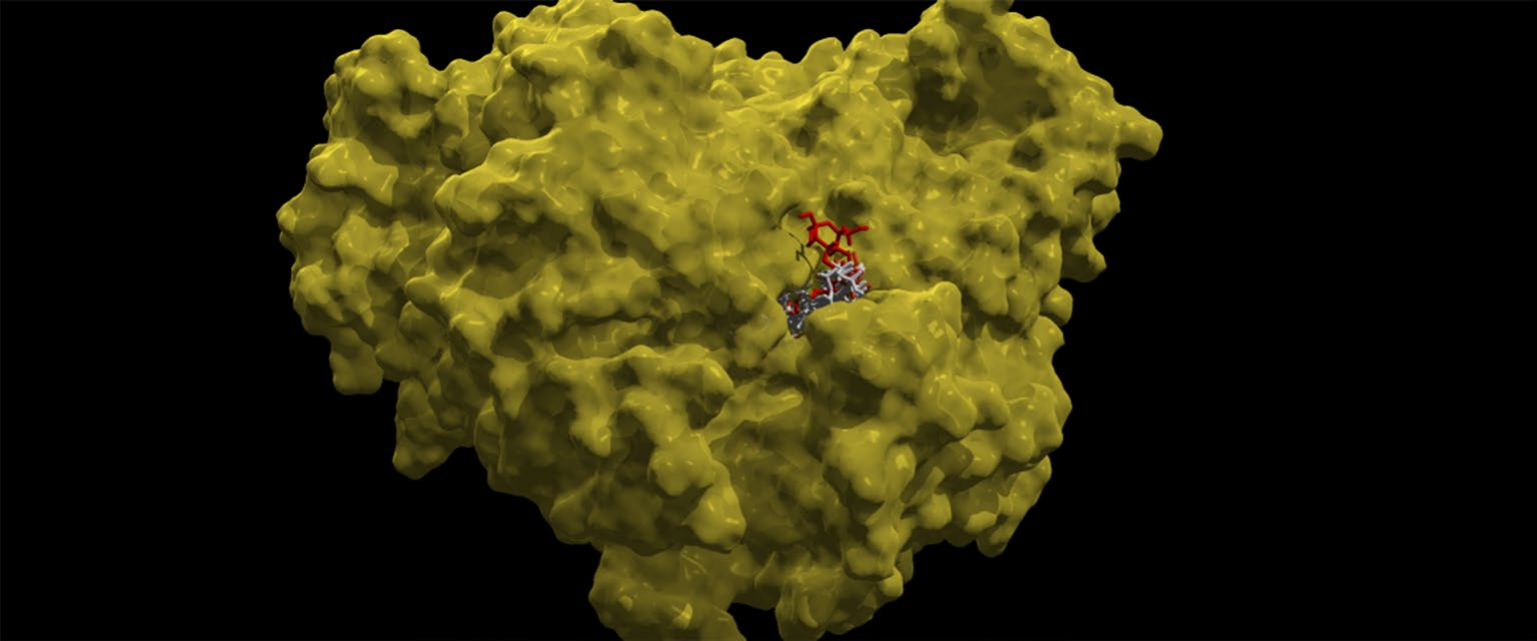
Superposition of docking poses of compounds **1**–**5** (in *white*) and acarbose (in *red*)

**Fig. 4:**
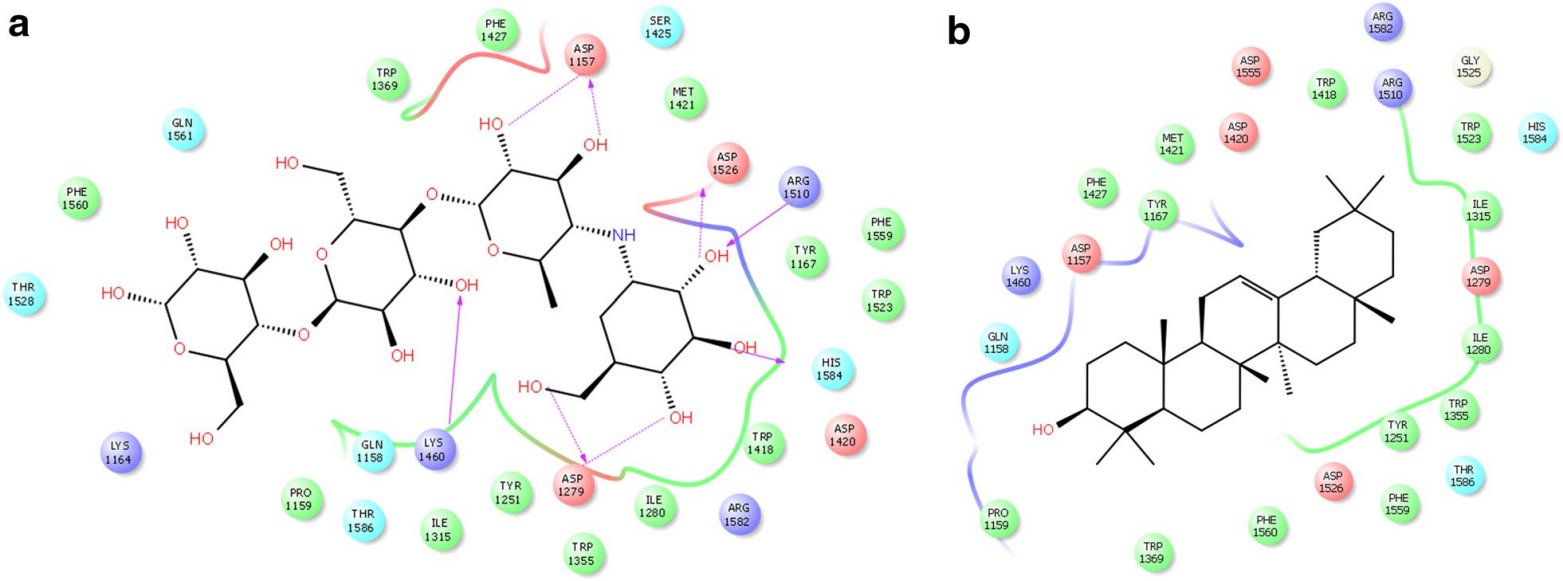
Comparision between the interaction of acarbose (**a**) and compound **2** (**b**) with glucosidase active site

**Fig. 5:**
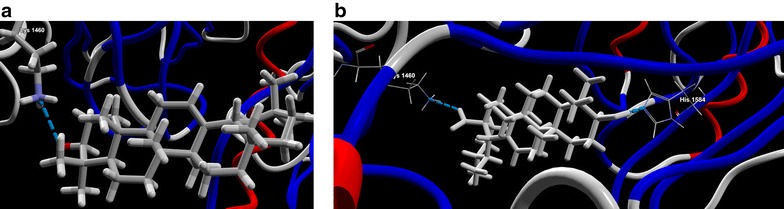
Docking poses of compound **2** (**a**) and compound **3** (**b**). Interaction with Lys 1460 is *highlighted*

Taking into account the preliminary in vitro α-glucosidase inhibition evaluations, the most active compounds (pentacyclic triterpenes) were selected to explain the experimental activities. Based on this, molecular docking study was conducted to evaluate the putative binding mode of compounds **1**–**5** into the human intestinal α-glucosidase (PDB: 3TOP). Results indicate that all analyzed triterpenoid compounds bind mainly through hydrophobic interactions. Figure 3 shows the superposition of docking poses of compounds **1**–**5** and acarbose in the binding site. It is interesting to note that despite analyzed triterpenoids are mainly hydrophobic, they bind into the same site of acarbose that is a more polar compound. As expected, acarbose interacts with the binding site through many hydrogen bonds (Fig. 4a) and compounds **1**–**5** interact via hydrophobic interactions (Fig. 4b). However, all triterpenoids interact with Lys 1460 through formation of hydrogen bonds with 3β-OH group or via ionic interaction between the carboxylate group in the triterpenoid and the amine group of Lys 1460 (Fig. 5a, b). This interaction has some significance for enzyme inhibition since docking poses where the 3β-OH-Lys hydrogen bond interaction was present had lower scores than docking poses with the carboxylate-Lys ionic interaction (see Table 2). In fact, docking poses with lower scores for compound **5** always displayed an ionic interaction with Lys 1460. Poses of compound **5** have fewer hydrophobic interactions than poses of compounds **1**–**4**, and this could be a plausible explanation for its lower enzyme inhibition in comparison with the other compounds analyzed. Therefore, taking compound **5** as an outlier, there is a slight correlation between docking score and experimental IC_50_.

**Table 2:** Rerank scores obtained during docking studies

Compound	IC_50_ (mM)	Rerank score	Interaction with Lys 1420
**1**	1.45	33.4917	Hydrogen bond
**2**	0.02	−55.6135	Hydrogen bond
**3**	1.08	−6.54481	Ionic
**4**	0.98	−24.732	Hydrogen bond
**5**	2.37	−14.5051	Ionic
	r^2^	0.3195	
	r^2^ (**5** as outlier)	0.8642	

## Conclusions

In summary, ten compounds were isolated from the endemic mangrove *P. rhizophorae* (five triterpenes, three flavonols and two dithiolanes). Dithiolane is the only core of the three evaluated lacking antiparasitic activity. Even though all compounds reported in this work have been reported from other sources, this is the first report of secondary metabolites produced by *P. rhizophorae*. There have been few new options for the treatment of neglected tropical diseases in half a century, therefore it is important to search for new sources of antiparasitic compounds to help global programs aimed at combating neglected diseases. *P. rhizophorae* represents a new natural source against parasitic protozoans which produces several compounds with selective activity (compounds **1**, **4**, **5**, **8** and **10**) and low levels of cytotoxicity. In addition, five pentacyclic triterpenes with potent α-glucosidase inhibitory activity were isolated. These compounds exhibited a competitive type of inhibition against *S. cerevisiae* α-glucosidase. All triterpenes showed higher inhibitory activity than acarbose. Definitely, in vitro inhibitory properties against α-glucosidase enzyme are far superior to the antiparasitic properties of this plant. Therefore this plant might also be an interesting alternative for reducing levels of blood sugar of people affected by DM.

## Methods

### General experimental procedures

Melting point measurements were carried out on an Electrothermal apparatus and are uncorrected. NMR spectra were acquired on Jeol Eclipse 400 MHz. APCI-HR-MS were acquired on a JEOL LC-mate mass spectrometer. The purification of the compounds was carried out on Agilent 1100 HPLC system equipped with a quaternary pump, a diode array detector and a reverse phase silica gel column (Phenomenex Synergy Hydro-RP, 250 mm × 100 mm, 4 μm) or normal phase silica gel column (Sphereclone silica 250 × 10 mm column) at a flow rate of 1.0 mL/min. TLC was performed on precoated silica gel 60 F254 plates (Merck). All solvents were HPLC grade and used without further purification [3, 28].

### Plant material and extract preparation

*P. rhizophorae* (Pellicieraceae) leaves were collected at Punta Chame, Panama, in May 2012. This plant was identified by Alejandro De Sedas. A voucher specimen (105833) has been deposited at the University of Panama Herbarium. After drying the fresh leaves and crushing in a standard blender to obtain 108 g of coarse powder, the extract was prepared by maceration with a mixture of MeOH:CHCl_3_ (1:1). Extract was concentrated to a semisolid paste using a Buchi Rotary Evaporator (R-215) to obtain 31 g of crude extract.

### Isolation of compounds

The organic extract was fractionated by column chromatography on silica gel (100 g). The column was eluted with hexane, followed by a gradient of hexane:EtOAc (1:0 → 0:1) and finally with a gradient of EtOAc:MeOH (1:0 → 1:1). Altogether, 325 fractions (100 ml each) were collected and combined according to their TLC profiles to yield 37 primary fractions (FI to FXXXVII). Fraction FIX (5.77 g) was further subjected to silica gel column chromatography and eluted with a gradient of hexane:EtOAc (1:0 → 0:1). This process led to 17 secondary fractions (FIX-A to FIX-Q). Fraction FIX-D (407 mg) eluted with Hexane:EtOAc (8:2), was purified by normal phase HPLC (Sphereclone silica 250 × 10 mm column, isocratic elution of 90 % hexanes:10 % EtOAc, UV detector at 254 nm, flow of 1 mL/min) to afford 21 mg of α-amyrine (**1**) and 13 mg of β-amyrine (**2**). Fraction FXIII (773 mg) eluted with Hexane:EtOAc (1:1), purified by normal phase HPLC (Sphereclone silica 250 × 10 mm column, isocratic elution of 85 % hexanes:15 % EtOAc, UV detector at 254 nm, flow of 1 mL/min) yielded 37 mg of ursolic acid (**3**), 27 mg of oleanolic acid (**4**) and 7 mg of betulinic acid (**5**). Fraction FXXVIII (1.78 g) was further subjected to silica gel column chromatography and eluted with a gradient of hexane:EtOAc (1:1 → 0:1) and EtOAc:MeOH (1:0 → 1:1). This process led to six secondary fractions (FXXVIII-A to FXXVIII-F). Fraction FXXVIII-A (98 mg) eluted with hexane:EtOAc (1:1) was purified by normal phase HPLC (Sphereclone silica 250 × 10 mm column, isocratic elution of 25 % hexanes:75 % EtOAc, UV detector at 254 nm, flow of 1 mL/min) resulted in 2 mg of brugierol (**6**) and 1.3 mg of iso-brugierol (**7**). Fraction FXXVIII-C (165 mg) eluted with 100 % EtOAc, was purified by reverse phase HPLC (Synergi Hydro-RP 250 × 10 mm column, isocratic elution of 40 % MeOH:60 % H_2_O, UV detector at 254 nm, flow of 1.0 mL/min) to afford 3.4 mg of Kaempferol (**8**) and 5.0 mg of quercetin (**9**). Finally, fraction FXXXIII (95 mg), eluted with EtOAc:MeOH (1:1), was purified by reverse phase HPLC (Synergi Hydro-RP 250 × 10 mm column, isocratic elution of 65 % MeOH:35 % H_2_O, UV detector at 254 nm, flow of 1.0 mL/min) yielded 8 mg of quercetrin (**10**).

### Spectral compounds data

*α-amyrin (****1****)* Colorless solid. m.p. 185–187 °C. ^1^H-NMR (CDCl_3_, 400 MHz): δ_H_ 5.16 (t, *J* = 3.6 Hz), 3.23 (dd, *J* = 4.4, 3.9 Hz), 1.96 (td, *J* = 4.4, 13.6 Hz), 1.85 (m), 1.78 (td, *J* = 4.9, 13.6 Hz), 1.00 (s), 0.97 (s), 0.94 (s), 0.87 (s), 0.83 (d, *J* = 6.0 Hz), 0.79 (sb) 0.74 (d, *J* = 11.0 Hz). ^13^C-NMR (100 MHz, CDCl_3_): δ_C_ 38.8 (C-1), 28.6 (C-2), 79.3 (C-3), 38.8 (C-4), 55.2 (C-5), 18.3 (C-6), 32.4 (C-7), 40.6 (C-8), 47.7 (C-9), 36.9 (C-10), 23.3 (C-11), 124.4 (C-12), 139.6 (C-13), 42.1 (C-14), 27.3 (C-15), 26.6 (C-16), 33.7 (C-17), 59.1 (C-18), 39.6 (C-19), 39.7 (C-20), 31.2 (C-21), 41.5 (C-22), 28.1 (C-23), 15.7 (C-24), 15.6 (C-25), 16.8 (C-26), 23.3 (C-27), 28.1 (C-28), 17.5 (C-29), 21.4 (C-30). APCI-HR-MS *m/z* 427.3893 [M + H]^+^ (calcd for C_30_H_51_O, 427.3895).

*β-amyrin (****2****)* Colorless solid. m.p. 196-197 °C. ^1^H-NMR (CDCl_3_, 400 MHz): δ_H_ 5.18 (t, *J* = 3.5 Hz), 3.20 (dd, *J* = 4.4, 10.8 Hz), 1.90 (td, *J* = 4.0, 13.6 Hz), 1.81 (m), 1.73 (td, *J* = 4.2, 13.6 Hz), 1.19 (s), 1.09 (s), 0.96 (s), 0.93 (s), 0.92 (d, *J* = 6.4 Hz), 0.84 (s), 0.80 (s), 0.72 (d, *J* = 10.8 Hz). ^13^C-NMR (100 MHz, CDCl_3_): δ_C_ 38.6 (C-1), 27.2 (C-2), 79.0 (C-3), 38.8 (C-4), 54.9 (C-5), 18.4 (C-6), 32.6 (C-7), 39.8 (C-8), 47.7 (C-9), 36.8 (C-10), 23.5 (C-11), 121.7 (C-12), 145.2 (C-13), 41.7 (C-14), 26.1 (C-15), 27.2 (C-16), 32.5 (C-17), 47.3 (C-18), 46.8 (C-19), 31.2 (C-20), 34.7 (C-21), 37.1 (C-22), 28.1 (C-23), 15.6 (C-24), 15.7 (C-25), 16.9 (C-26), 25.8 (C-27), 28.4 (C-28), 33.7 (C-29), 23.7 (C-30). APCI-HR-MS *m/z* 427.3896 [M + H]^+^ (calcd for C_30_H_51_O, 427.3895).

*Ursolic acid (****3****).* Colorless solid. m.p. 291–292 °C. ^1^H NMR (CDCl_3_, 400 MHz): δ_H_ 5.28 (t, *J* = 3.6 Hz), 3.21 (dd, *J* = 10.2, 4.4 Hz), 2.18 (d, *J* = 11.7 Hz), 1.19 (m), 2.00 (dd, *J* = 13.0, 4.0 Hz), 1.25 (s), 0.98 (s), 0.77 (s), 1.08 (s), 1.14 (s), 0.93 (d, *J* = 6.5 Hz), 0.91 (d, *J* = 5.9 Hz). ^13^C NMR (CDCl_3_,100 MHz): δ_C_ 39.2 (C-1), 27.5 (C-2), 78.5 (C-3), 38.7 (C-4), 55.5 (C-5), 18.3 (C-6), 33.1 (C-7), 39.6 (C-8), 47.8 (C-9), 36.9 (C-10), 16.6 (C-11), 125.7 (C-12), 138.4 (C-13), 41.7 (C-14), 29.5 (C-15), 24.1 (C-16), 47.7 (C-17), 53.1 (C-18), 39.2 (C-19), 39.2 (C-20), 30.5 (C-21), 36.9 (C-22), 28.0 (C-23), 15.2 (C-24), 14.8 (C-25), 16.4 (C-26), 23.1 (C-27), 180.4 (C-28), 22.9 (C-29), 22.8 (C-30). APCI-HR-MS *m/z* 457.3635 [M + H]^+^ (calcd for C_30_H_49_O_3_, 457.3637).

*Oleanolic acid (****4****)* Colorless solid. UV (MeOH) m.p. 299–301 °C.; ^1^H NMR (CDCl_3_, 400 MHz): δ_H_ 5.24 (t, *J* = 3.6 Hz), 3.21 (dd, *J* = 10.2, 4.4 Hz), 2.82 (dd, *J* = 12.7, 4.3 Hz), 0.96 (s), 0.78 (s), 0.84 (s), 0.76 (s), 1.25 (s), 0.87 (s), 0.93 (s). ^13^C NMR (CDCl_3_, 100 MHz): δ_C_38.6 (C-1), 26.7 (C-2), 78.5 (C-3), 39.2 (C-4), 55.5 (C-5), 18.3 (C-6), 32.6 (C-7), 39.6 (C-8), 48.1 (C-9), 37.0 (C-10), 22.7 (C-11), 122.4 (C-12), 144.1 (C-13), 42.0 (C-14), 27.7 (C-15), 22.8 (C-16), 46.7 (C-17), 41.5 (C-18), 46.1 (C-19), 30.4 (C-20), 33.7 (C-21), 32.3 (C-22), 28.8 (C-23), 14.7 (C-24), 15.1 (C-25), 16.5 (C-26), 25.2 (C-27),180.4 (C-28), 32.8 (C-29), 23.3 (C-30). APCI-HR-MS *m/z* 457.3639 [M + H]^+^ (calcd for C_30_H_49_O_3_, 457.3637).

*Betulinic acid (****5****)* Colorless solid. m.p. 317–319 °C. ^1^H NMR (CDCl_3_, 400 MHz): δ_H_ 4.66 (s), 3.79 (dd, *J* = 10.0, 5.5 Hz), 2.39 (m), 2.10–2.20 (m) 1.66 (s), 1.00 (s), 0.96 (s), 0.94 (s), 0.80 (s), 0.74 (s). ^13^C NMR (CDCl_3_, 100 MHz): δ_C_ 38.9 (C-1), 27.8 (C-2), 79.0 (C-3), 38.7 (C-4), 55.5 (C-5), 18.3 (C-6), 34.0 (C-7), 40.9 (C-8), 50.4 (C-9), 37.3 (C-10), 20.7 (C-11), 25.2 (C-12), 37.3 (C-13), 42.7 (C-14), 30.1 (C-15), 29.3 (C-16), 56.5 (C-17), 46.4 (C-18), 49.1 (C-19), 150.4 (C-20), 29.8 (C-21), 34.1 (C-22), 28.0 (C-23), 15.4 (C-24), 16.0 (C-25), 16.1 (C-26), 14.8 (C-27), 180.0 (C-28), 109.6 (C-29), 19.1 (C-30). APCI-HR-MS *m/z* 457.3636 [M + H]^+^ (calcd for C_30_H_49_O_3_, 457.3637).

*Quercetin (****6****)* Yellow powder, m.p. 313–315. ^1^H NMR (DMSO-D_6_, 400 MHz) δ_H_ 6.19 (d, *J* = 2.0, H-6), 6.41 (d, *J* = 2.0, H-8), 7.69 (d, *J* = 2.2, H-2′), 6.89 (d, *J* = 8.5, H-5′), 7.55 (d, *J* = 8.5, 2.2, H-6′), 12.98 (1H, s, 5-OH). ^13^C NMR (DMSO-D_6_, 100 MHz) δ_C_ 145.1 (C-2), 135.8 (C3), 175.9 (C-4), 103.1 (C-4a), 160.8 (C-5), 98.3 (C-6), 164.0 (C-7), 93.41 (C-8), 156.2 (C-9), 122.0 (C-1′), 115.10 (C-2′), 146.9 (C-3′), 147.8 (C-4′), 115.7 (C-5′), 120.1 (C-6′). APCI-HR-MS *m/z* 303.0569 [M + H]^+^ (calcd for C_15_H_10_O_7_, 303.0505).

*Kaempferol (****7****)* Pale yellow needles, m.p. 276–278 °C. ^1^H NMR (DMSO-D_6_, 400 MHz) δ_H_ 6.19 (d, *J* = 1.9, H-6), 6.45 (d, *J* = 1.9, H-8), 8.04 (d, *J* = 8.9, H-2′), 6.93 (d, *J* = 8.9, H-3′), 6.93 (d, *J* = 8.9, H-5′), 8.04 (d, *J* = 8.9, H-6′), 9.35 (br s, 3-OH), 10.10 (br s, 4-OH), 12.48 (br s, 5OH), 10.85 (s, 7-OH). ^13^C NMR (DMSO-D_6_, 100 MHz) δc: 148.1 (C-2), 137.3 (C-3), 177.6 (C-4), 104.5 (C-4a), 162.8 (C-5), 99.3 (C-6), 165.5 (C-7), 94.5 (C-8), 160.7 (C-8a), 123.9 (C-1), 130.9 (C-2), 116.5 (C-3), 158.3 (C-4), 116.4 (C-5), 130.9 (C-6) APCI-HR-MS *m/z* 287.0606 [M + H]^+^ (calcd for C_15_H_11_O_6_, 287.0556).

*Quercetrin (****8****)* Amorphous yellow powder, m.p. 181–182 °C, ^1^H NMR (DMSO-D_6_, 400 MHz): δ_H_ 7.36 (dd, *J* = 1.8, 8.1 Hz), 7.30 (d, *J* = 1.8 Hz), 6.87 (d, *J* = 8.4 Hz), 6.40 (d, *J* = 1.8 Hz), 6.21 (d, *J* = 2.2 Hz), 5.25 (d, *J* = 1.5 Hz), 4.20 (m), 3.73 (dd, *J* = 3.4, 9.2 Hz), 3.38 (m), 3.31 (m), 0.90 (d, *J* = 6.6 Hz); ^13^C NMR (DMSO-D_6_, 100 MHz): δ_C_ 178.1 (C-4), 164.8 (C-7), 161.9 (C-5), 157.5 (C-9), 157.0, (C-2) 149.0 (C-3′), 145.8 (C-4′), 134.8 (C-3), 121.6 (C-1′), 121.2 (C-6′), 116.2 (C-2′), 116.0 (C-5′), 104.7 (C-10), 102.4 (C-1″), 99.2 (C-6), 94.2 (C-8), 71.5 (C-5″), 71.2 (C-3″), 70.9 (C-2″), 70.6 (C-4″), 18.1 (C-6″); APCI-HR-MS *m/z* 449.1079 [M + H]^+^, C_21_H_21_O_11_ calcd for 449.1084).

*Brugierol (****9****)* Colorless solid, m.p. 88–89 °C, ^1^H NMR (CD_3_Cl, 400 MHz): δ_H_ 5.23 (bs, H-4), 3.74 (dd, *J* = 11.0, 6.0, H-5b), 3.53 (dd, *J* = 12.0, 6.0, H-3a), 3.41 (dd, *J* = 12.0, 2.1, H-3b), 3.34 (dd, *J* = 11.0, 6.0, H-5a). ^13^C (CD_3_Cl, 100 MHz): δ_C_ 43.3 (C-3), 75.8 (C-4), 70.0 (C-5). APCI-HR-MS *m/z* 139.9770 [M + H]^+^ (calcd for C_3_H_7_O_2_S_2_, 139.9767).

*Iso-brugierol (****10****)* Colorless solid, m.p. 82–83 °C, ^1^H NMR (CD_3_Cl, 400 MHz): δ_H_ 4.71 (bs, H-4), 4.10 (dd, *J* = 10.5, 1.0, H-5b), 4.08 (dd, *J* = 13.0, 1.0, H-3b), 3.63 (dd, *J* = 10.5, 3.8, H-5a), 3.02 (dd, *J* = 13.0, 3.8, H-5b). ^13^C (CD_3_Cl, 100 MHz): δ_C_ 48.1 (C-3), 80.2 (C-4), 67.8 (C-5). APCI-HR-MS *m/z* 139.9764 [M + H]^+^ (calcd for C_3_H_7_O_2_S_2_, 139.9767).

### Culture procedure

Promastigotes cultures of *L. donovani* are maintained in continuous log phase growth in Liver Infusion Tryptose (LIT) Medium, pH 7.2, supplemented with 10 % Fetal Bovine Serum (FBS) at 28 °C. For promastigote transformation into amastigote forms, 1 mL of promastigotes log phase culture is transferred into 5 mL of Medium 199 Modified (SIGMA-Cat M3769) pH 5.5, supplemented with 0.1 g/L l-glutamine, 2.2 g/L sodium bicarbonate, 2 g Glucose, 5 mL penicillin–streptomycin and 20 % FBS and maintained at 35 °C until its use for bioassays.

Antiplasmodial activity is evaluated using a chloroquine-resistant strain (Indochina W2) of *P. falciparum*. The cultures are kept in synchrony by thermal cycling incubation [29] and are maintained in continuous log phase growth in RPM-I1640 medium (SIGMA) supplemented with 2 % washed human O Rh positive (+) erythrocytes, 25 mM HEPES, 32 nM NaHCO_3_, and 10 % Human Serum from an O Rh+ donor. All cultures and assays are conducted at 37 °C under an atmosphere of 5 % CO_2_ and 5 % O_2_, with a balance of N_2_ (90 %).

*T. cruzi* (Tulahuen) C4 strain lactosidase (Lac Z) gene [30]. The strain is maintained on VERO Cells (African Green Monkey cell line obtained from ATCC on 2006), grown in monolayers in RPM-I1640 medium, supplemented with 10 % heat inactivated FBS. All cultures and assays are conducted at 37 °C under an atmosphere of 5 % CO_2_/95 % air mixture.

### In vitro antiparasitic assays

Dry samples were diluted in 100 % DMSO (dimethylsulfoxide) to obtain a concentration of 4 mg/mL. Samples are used immediately in the bioassay and stored at −20 °C in the dark until results are obtained. All assays are performed in sterile 96-well microtitre culture plates (Costar Cat 3595). In a primary screening samples are tested in duplicate wells at a final concentration of 10 μg/mL. If activity is found (growth Inhibition >75 % for *L. donovani* and *P. falciparum* and growth Inhibition >50 % for *T. cruzi*) then an assessment of the concentration that inhibits 50 % of growth (IC_50_) is carried out with four concentrations, by duplicates. The IC_50_ is analyzed with the Excel Add-On software LSW Data Analysis Tool. The concentration that inhibited the growth of the parasites to 50 % (IC_50_) was calculated through the inhibition curve of the obtained optical density values, and compared to the untreated controls.

*L. donovani* and *P. falciparum* A DNA cross linking agent is used to determine the amount of parasites in culture. After 48 and 72 h incubation, respectively, 1 % PicoGreen® solution is added to all wells in the dark. After shaking, the plate is taken into a microplate reader employing 485/20 nm excitation and 528/20 nm emission filter sets. Amphotericin B was used as a positive control for Leishmania; the typical IC_50_ response of *L. donovani* to this drug is 70–120 ng/μl. Chloroquine served as a positive control for *P. falciparum* (IC_50_ = 80–100 nM) [3].

*T. cruzi* In this assay, a colorimetric method is used to determine the inhibition of parasite growth as detected by reduction of β-galactosidase (β-Gal) as a reporter gene, expressed by the Tulahuen clone C4 of *T. cruzi* [22]. Assays are performed on trypomastigotes, the intracellular form of the parasite infecting African green monkey kidney (VERO) cells, exposed during 120 h to different concentrations (50, 10 and 2 µg/mL) of the test substance. The resulting colour from the cleavage of chlorophenol red-β-d-galactoside (CPRG) by β-Gal expressed by the parasite, was measured using a Benchmark Bio-Rad microplate reader at 570 nm. Nifurtimox was used as a positive control (IC_50_ 0.15–13.4 µM) [3, 31].

### Cytotoxicity bioassay

Vero cells were seeded in 96-well plates in RPM-I1640 medium supplemented with 10 % FBS and 1 % penicillin/streptomycin. The cells were allowed to grow for 24 h before adding the test compounds, dissolved in DMSO, to final concentrations of 10, 4, 0.2 and 0.08 µg/mL. A sample with only a volume of DMSO similar to the added volume in the compounds samples was placed as a negative control in all plates. All samples were incubated for five days before staining and examining for reduction of 3-(4,5-dimethylthiazol-2-yl)-2,5-diphenyltetrazolium bromide (MTT) and analyzed 4 h later in a color plate reader at 570 nm.

### α-Glucosidase inhibitory assay

The α-glucosidase inhibitory assay was performed according to Chan and collaborators (2010) [32], with modifications. α-glucosidase from baker’s yeast purchased from Sigma Chemical Co. The inhibition was measured spectrophotometrically at pH 7.0 and 37 °C employing 2 mM p-nitrophenyl α-d-glucopyranoside (PNP-G) as a substrate and 32 mU/mL of enzyme, in 100 mM potassium phosphate buffer (enzyme stock). Acarbose was dissolved in phosphate buffer, and serial dilutions (in order to obtain the IC_50_) were prepared and employed as positive control. The absorbance (A) of 4-nitrophenol released by the hydrolysis of PNP-G was measured at 400 nm by Synergy HT Bio Tek microplate spectrophotometer. A 20 µL of acarbose or test compounds solution was incubated for 7 min with 150 µL of enzyme stock at 37 °C. After incubating, 150µL of substrate was added and further incubated for 20 min at 37 °C. All assays are performed in 96-well microplates (Greiner bio-one 655101) in duplicate. The activity of samples was calculated as a percentage in comparison to a control (DMSO or MeOH instead of sample solution) according with the following equation:

$${\% }Inhibition = \left( (\Delta \;A_{control} - \Delta\; A_{sample})/{\Delta \;A_{control} } \right) \times 100\;\%$$

The concentration required to inhibit activity of the enzyme by 50 % (IC_50_) was calculated by regression analysis [33].

### Docking study

All ligands were constructed in Spartan’10 [34], and their geometry was optimized using MMFF force field. Protein–ligand docking studies were carried out based on the crystal structures for C-terminal domain of human intestinal α-glucosidase (PDB: 3TOP) [35] which was retrieved from the Protein Data Bank [36]. Prior to docking, all of the solvent molecules and the co-crystallized ligand was removed. Molecular docking calculations were performed using Molegro Virtual Docker v. 6.0.1 [37]. A sphere of 15 Å radius was centered in the binding site for searching. Experimental data indicates that these compounds are competitive inhibitors; thus the active site was chosen as the binding site. Protonation states and assignments of the charges on each protein were based on standard templates as part of the Molegro Virtual Docker program, and no other charges were necessary to set. Flexible ligand models were used in the docking and subsequent optimization scheme. Different orientations of the ligands were searched and ranked based on their energy scores. The RMSD threshold for multiple cluster poses was set to <1.00 Å. The docking algorithm was set to 5000 maximum iterations with a simplex evolution population size of 100 and a minimum of 50 runs for each ligand. After docking, a number of further scores were calculated including the binding affinity (MolDock Score) and re-ranking score (Rerank Score). The re-ranking score utilizes a more advanced scoring scheme than that used during docking and is often more useful for accurate ranking of the poses. Poses with lower score were selected for further analysis

To assess the efficacy of this procedure for finding low energy solutions, the co-crystallized ligand (acarbose) was also docked. The top ranking score was recorded, and the RMSD of that pose from the corresponding crystal coordinates calculated. RMSD was lower than 2Å, indicating that the methodology used in the molecular docking simulation is appropriate.
